# Strong Coupling of Folded Phonons with Plasmons in 6H-SiC Micro/Nanocrystals

**DOI:** 10.3390/molecules23092296

**Published:** 2018-09-08

**Authors:** Yao Huang, Run Yang, Shijie Xiong, Jian Chen, Xinglong Wu

**Affiliations:** 1 National Laboratory of Solid State Microstructures and School of Physics, Nanjing University, Nanjing 210093, China; huangyao11@huawei.com (Y.H.); sjxiong@nju.edu.cn (S.X.); 2 National Laboratory of Solid State Microstructures and Research Institute of Superconductor Electronics, Nanjing University, Nanjing 210093, China; chenj63@nju.edu.cn

**Keywords:** SiC micro/nanocrystals, plasmon–phonon coupling, folded phonons

## Abstract

Silicon carbide (SiC) has a large number of polytypes of which 3C-, 4H-, 6H-SiC are most common. Since different polytypes have different energy gaps and electrical properties, it is important to identify and characterize various SiC polytypes. Here, Raman scattering is performed on 6H-SiC micro/nanocrystal (MNC) films to investigate all four folded transverse optic (TO) and longitudinal optic (LO) modes. With increasing film thickness, the four folded TO modes exhibit the same frequency downshift, whereas the four folded LO modes show a gradually-reduced downshift. For the same film thickness, all the folded modes show larger frequency downshifts with decreasing MNC size. Based on plasmons on MNCs, these folded modes can be attributed to strong coupling of the folded phonons with plasmons which show different strengths for the different folded modes while changing the film thickness and MNC size. This work provides a useful technique to identify SiC polytypes from Raman scattering.

## 1. Introduction

Silicon carbide (SiC) is a wide bandgap semiconductor having many applications such as high-temperature and high-voltage power electronics, functional ceramics, biosensing, and 3D printing due to the excellent thermal conductivity, high breakdown filed strength, and high saturated carrier velocity [1,2,3,4]. It is also known to have a large number of polytypes of which the 3C-, 4H-, 6H-SiC are most common [5,6,7]. Since different polytypes have different energy gaps and electrical properties [8,9], it is important to identify and characterize the various SiC polytypes and the structures, and a powerful technique for achieving this is Raman scattering [10,11,12,13,14,15].

According to the group-theoretical selection rule, there are additional phonon absorption lines for all polytypes, for example, eight in 6H-SiC [16]. To avoid the complexity caused by various phonon branches, the concept of standard large zone and folded modes has been adopted [10,11,12,13,14,15,16,17]. In 6H-SiC, the reduced momentum value x (x = q/q_max_ = 0, 1/3, 2/3, and 1, q_max_ = 6π/a) can be measured [17]. Hence, for the transverse optic (TO) and longitudinal optic (LO) phonon branches, there are four TO and four LO modes at most. With the exception of the TO and LO modes at x = 0, all the others are the folded modes. Compared to the TO and LO modes, the planar and axial ones are more appropriate to describe these fold modes and, for convenience, the terms of folded TO (FTO) and folded LO (FLO) are used to represent the folded planar/axial optic modes [18]. These folded modes are very weak because their dipole moments arise from very small site differences in the polytype stacking arrangements [19]. The folded effect of Raman modes is different in different SiC polytypes. The Raman modes of pure 6H-SiC crystals have been determined experimentally by Feldman et al. [16], but the FLO modes with the B_1_ symmetry at x = 1/3 and 1 have not been observed because they are not Raman or infrared active. Hence, the doublet structure of the FLO mode with A_1_ symmetry at x = 2/3 and FTO mode with E_2_ symmetry at x = 1/3 remain unresolved. It has recently been found that 3C-SiC nanocrystals can have plasmons due to surface conversion from *sp*^3^ to *sp*^2^ hybridization which affects the intensity of the LO mode [20,21]. Xu et al. have also reported that in the presence of the phonon–plasmon coupling effect at the interface of 3C-SiC/metal oxide microcrystals (MNCs), the TO modes of 3C-SiC can downshift (Raman red-shift) by about 6 to 15 cm^−1^ [21]. Particularly, recent reports have also pointed out that SiC nanocrystals or nanowires have obvious surface plasmon effect and thus are of various favorable applications such as in biological/chemical sensing, efficient and robust oxygen evolution [22,23,24]. These phenomena provide clues that plasmon coupling may also affect the weak folded modes in SiC polytype crystals making the unobserved folded modes visible.

In this work, 6H-SiC micro/nanocrystal (MNC) films with different MNC sizes and film thicknesses are prepared to investigate the Raman scattering characteristics. Some weak and unobserved folded modes are enhanced and become observable due to strong coupling of these folded phonons with the plasmons of 6H-SiC MNCs. In addition, the phonon-plasmon coupling leads to observable frequency downshift of the folded phonon modes to different extents, depending on the MNC size and film thickness.

## 2. Results

**Figure 1:**
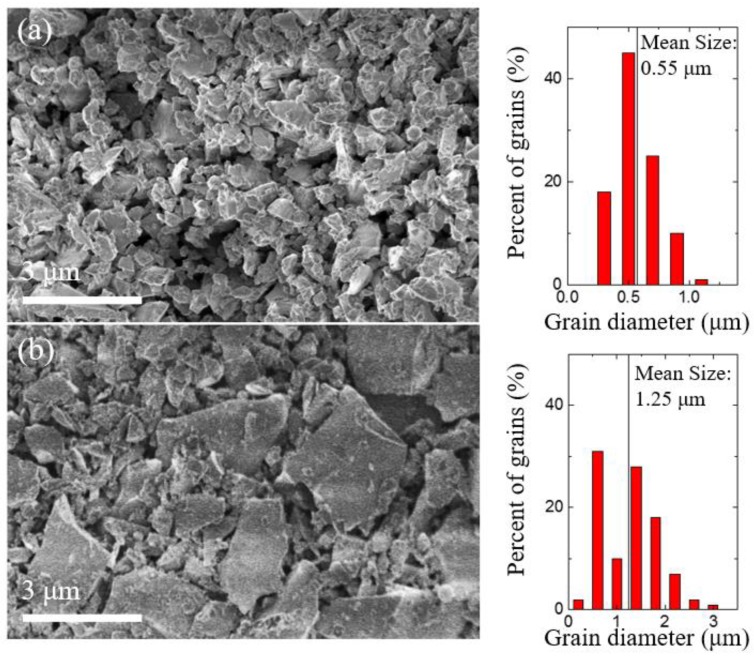
Scanning electron microscopy (SEM) images (left side) of the 6H-SiC films with a mean size of (**a**) 0.55 μm and (**b**) 1.25 μm. The right side shows the corresponding micro/nanocrystal (MNC) size distributions.

**Figure 2:**
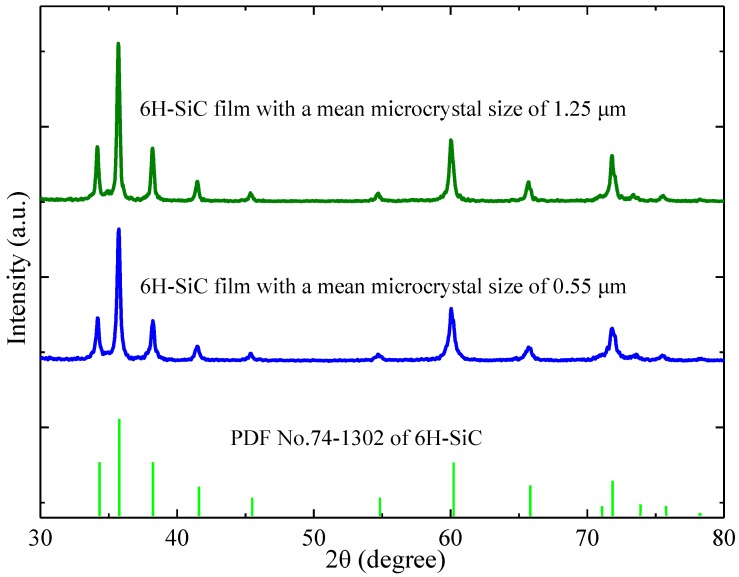
X-ray diffraction (XRD) patterns acquired from the 6H-SiC films with mean sizes of 1.25 and 0.55 μm. The XRD pattern of the standard 6H-SiC crystal is also presented for comparison.

Figure 1 shows the scanning electron microscopy (SEM) images of two typical 6H-SiC MNC films and MNC size distributions (right sides). The SiC films with MNC sizes of 400–600 nm have more concentrated grain diameters at a mean value of 0.55 μm (Figure 1a). The other SiC MNC film with a mean size of 1.25 μm shows a slightly nonuniform MNC size distribution (Figure 1b). To determine the SiC polytype, X-ray diffraction (XRD) is performed and, as shown in Figure 2, all the diffraction peaks from the two SiC films can be indexed to the standard 6H-SiC crystal, indicating that the films contain only 6H-SiC MNCs.

**Figure 3:**
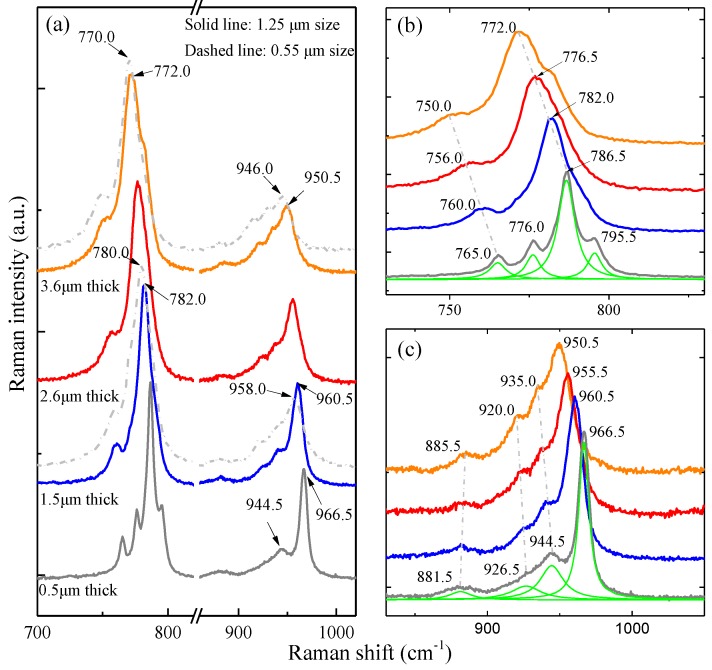
(**a**) Raman spectra of the 6H-SiC films with mean sizes of 1.25 μm (solid lines) and 0.55 μm (dashed lines) but different thicknesses; (**b**,**c**) Magnified spectral regions of the folded transverse optic (FTO) and folded longitudinal optic (FLO) bands in which the Lorenzian line-shape is adopted to fit the Raman spectra of the 0.5 μm thick films.

For long-wavelength phonons, only the axial direction of the large zone needs to be taken into account [25]. In 6H-SiC, the standard large zone is q_max_ = 6π/*a*, where *a* is the axial dimension of the basic cell. Here, the concepts of pseudomomentum vector and reduced momentum are adopted [26]. As 2π/*a* is a reciprocal vector, if the pseudomomentum vector q_max_ = 6π/*a*, reduced momentum x = q/q_max_, and x = 0, 1/3, 2/3, and 1, the reduced momentumin the large zone are almost equal to zero in the Brilliouin zone. Through surface modification by ethanol during the preparation process, all the eight theoretically predicted phonon modes can be seen from the Raman spectra (see Supporting Information). The Raman spectra of the 6H-SiC films with a mean size of 1.25 μm and four different thicknesses are displayed in Figure 3a, in which two Raman spectra of the 6H-SiC films with a mean size of 0.55 μm and thicknesses of 1.5 and 3.6 μm are shown for comparison. With regard to the 0.5 μm thick SiC film, the peaks at 795.5, 786.5, and 765.0 cm^−1^ are assigned to the FTO modes at x = 0, 1/3, and 1 (Figure 3b) [27,28,29]. There is a new peak at 776.0 cm^−1^ between the x = 1/3 and 1 FTO modes, which can be assigned to the x = 2/3 FTO mode. The four folded modes which show noticeable intensities can be identified clearly. In the LO phonon region of 900–1000 cm^−1^, the 966.5 cm^−1^ peak shows the strongest intensity and can be attributed to x = 0 [30,31]. The 944.5 cm^−1^ peak may be assigned to the FLO mode with x = 1/3. According to the enlarged figure of the FLO region (Figure 3c), there are two small bumps on the left side of the x = 1/3 FLO peak. The central positions of the two bumps are at 926.5 and 881.5 cm^−1^ with Lorenzian fitting. The two peaks can be observed more clearly from the thicker SiC films and the intensity enhancement arises from FLO-plasmon coupling which becomes stronger when the film is thicker. They should belong to the x = 2/3 and 1 FLO modes.

Figure 3a shows that the film thickness affects the mode frequency. With increasing film thickness, the four FTO/FLO modes show obvious frequency downshifts. If we use the Lorenzian line-shape to fit these peaks, the two well-resolved FTO modes at 795.5 and 776.0 cm^−1^ (x = 0 and 2/3) of the 0.5 μm thick film become the right and left shoulders of the strongest FTO mode at 786.5 cm^−1^ (x = 1/3) (Figures S1–S4), but the FTO mode at 765.0 cm^−1^ (x = 1) is still a well-resolved single peak (Figure 3b). For example, the strongest x = 1/3 FTO mode shifts to 782.0 cm^−1^ with a downshift of 4.5 cm^−1^ for the 1.5 μm thick film and to 772.0 cm^−1^ with a downshift of 14.5 cm^−1^ for the 3.6 μm thick film. The lowest x = 1 FTO mode shifts to 760.0 cm^−1^ with a downshift of 5 cm^−1^ for the 1.5 μm thick film and to 750.0 cm^−1^ with a downshift of 15.0 cm^−1^ for the 3.6 μm thick film. The downshift trend of the FLO mode is similar to that related to the film thickness (Figure 3c). The strongest x = 0 FLO mode shifts to 960.5 cm^−1^ with a downshift of 6.0 cm^−1^ for the 0.5 to 1.5 μm thick films and to 950.5 cm^−1^ for the 3.6 μm thick film with a downshift of 16.0 cm^−1^. The frequencies of all the FTO and FLO modes of the samples with different MNC size and film thickness are listed in Table 1. In the films with the same MNC size, the frequency downshift with increasing film thickness is the same for all the FTO modes, but it is different for the FLO modes, for example, 16.0 cm^−1^ for the x = 0 FLO mode and 9.5 cm^−1^ for the x = 1/3 FLO mode for the 0.5 to 3.6 μm thick films, respectively.

Figure 3a and Table 1 show that if the MNC size decreases from 1.25 to 0.55 μm and the film thickness remains the same, the FTO mode frequencies further downshift and the degree of downshift is the same for films with different thickness. However, for the FLO modes, the degree of downshift is different and becomes larger with increasing film thickness (dashed lines in Figure 3). For example, for the strongest x = 1/3 FTO mode, the downshift amount of the 1.5 and 3.6 μm thick films is the same (2.0 cm^−1^) for sizes of 1.25 and 0.55 μm (Figure S5). For the strongest x = 0 FLO mode, the downshift amount is 2.5 cm^−1^ for the 1.5 μm thick film and 4.5 cm^−1^ for the 3.6 μm thick film (Figure S6). Thus, the MNC size and film thickness both affect the FTO/FLO mode frequency downshifts. In addition, the frequencies of the FTO and FLO modes for the same film thickness show larger downshifts with decreasing MNC size.

**Figure 4:**
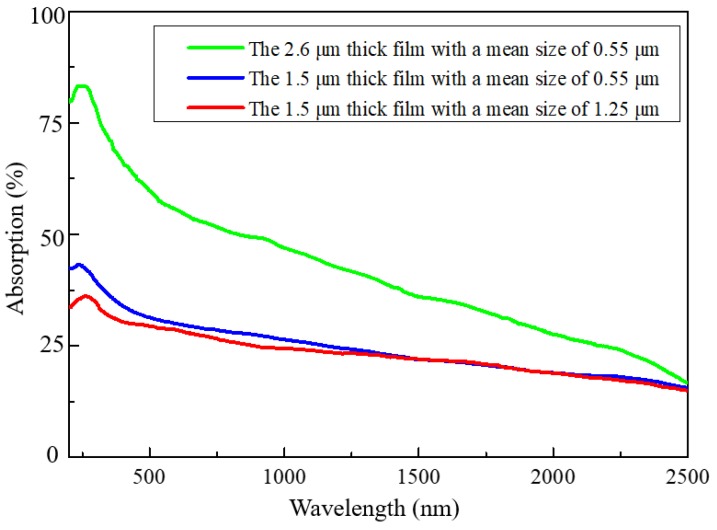
UV–VIS–NIR absorption of the 6H-SiC films with different thicknesses and MNC sizes. Due to the wide MNC size distribution, the absorption spectra are broad and have large intensities in the range of visible light. The strongest absorption appears in the film with the largest thickness and meantime with the smallest MNC sizes. The smaller the MNC sizes, the larger the specific surface area and thus the stronger the surface plasmons.

Based on previous studies [20,21,32,33,34,35], the observed downshifts of the folded modes may be caused by phonon-plasmon coupling in the 6H-SiC MNCs. The stronger the coupling to plasmons, the larger the frequency downshift should be. According to the Raman spectra, coupling to plasmons is enhanced for thicker films and smaller MNCs. Since the observed folded phonon modes are more likely to be bulk ones with moments as good quantum numbers, the plasmons coupled to them should also be the extended modes. Hence, the film surface is a hard-scattering source for them and scattering should shorten the lifetime of the modes and weaken coupling. With increasing film thickness, surface scattering is reduced and coupling to plasmons should be strengthened. On the contrary, the boundaries of MNCs are usually associated with vacancy defects of the crystals which can be regarded as the source of unintentionally doped free carriers. Thus, by increasing the MNC size, the density of defects decreases, and this weakens coupling between the phonons and plasmons. As reported previously [35], the classical dielectric function is represented by the sum of the contributions from phonons and free carriers and no direct interaction between them is taken into account. Therefore, only plasmons from high carrier concentration with frequencies near the phonon frequencies have effects on Raman spectra. However, in our present case, the plasmons stem from low carrier concentration related to the surface modification and thus their frequencies are much lower than those of the phonons. Here, the effective field of the plasmons acts on not only polarization of plasmons themselves but also effective polarization of the phonon modes. Such direct coupling can take place because of the existence of multiple folded phonon modes and the beat effect of system nonlinearity. In fact, the observed large frequency red-shift is clear evidence of this strong coupling. The acquired ultraviolet-visible-near-infrared (UV–VIS–NIR) absorption spectra also support a fact that the thicker film with smaller MNC sizes has stronger light absorption (Figure 4, green line). This is because the smaller the MNC sizes, the larger the specific surface area, which means that more light can be absorbed to produce stronger surface plasmons. Here we would like to point out that the MNC sizes with micrometer order cannot lead to obvious bandgap modification due to the reduced sizes. Thus, the absorption peak is almost at the same wavelength. 

## 3. Discussion

To theoretically explore the downshift mechanism of the FTO and FLO modes in the presence of plasmons, the Raman spectra are calculated by taking into account coupling of all the FTO and FLO modes with the plasmons. The polarizations of these modes and the plasmons, denoted as *P_TO_*_,*i*_*, P_LO_*_,*i*_ (*i* = 1, 2, 3, 4)*,* and *P_e_*, respectively, respond linearly to the local effective total fields *E_TO_*_,*i*_, *E_LO_*_,*i*_*,* and *E_e_* experienced by them:

(1) $$P_{LO(TO),i}=\chi_{l(t),i}E_{LO(TO),i},\;\mathrm{and}\;P_{e}=\chi_{e}E_{e}$$

Here, the frequency-dependent susceptibilities without coupling of the phonon modes with plasmons are given by [36]:

(2) $$4\pi \chi_{l(t),i}=\frac{\omega_{t,i}^{2}-\omega_{l,i}^{2}}{\omega_{l(t),i}^{2}-\omega^{2}-i\omega \Gamma_{l(t),i}}\varepsilon_{\infty },\;\mathrm{and}\;4\pi \chi_{e}=-\frac{\omega_{e}^{2}}{\omega (\omega +i\gamma )}\varepsilon_{\infty }$$

where *ε*_∞_ is the dielectric constant at the large frequency limit, *ω_l_*_(*t*),*i*_ and Γ*_l_*_(*t*),*i*_ are the frequency and damping rate of the *i*th FTO (FLO) mode, respectively, and *ω_e_* and *γ* are the frequency and damping rate of plasmons. In the presence of phonon–plasmon coupling, the effective total field is expressed as:

(3) $$E_{LO(TO),i}=D_{LO(TO),i}-4\pi P_{LO(TO),i}/\varepsilon_{\infty }+\eta_{LO(TO),i}P_{e},\;\mathrm{and}\;E_{e}=D_{e}-4\pi P_{e}/\varepsilon_{\infty }+\sum_{i}\left(\eta_{LO,i}P_{LO,i}+\eta_{TO,i}P_{TO,i}\right)$$

where *η_LO_*_(*TO*),*i*_ is the coupling strength of the *i*th LO (TO) mode with plasmons and *D_LO_*_(*TO*)*,i*_ and *D_e_* are the effective driving fields acting on the *i*th FLO (FTO) mode and plasmons, respectively. There are some common parameters, such as the film thickness and MNC size, in our experiments which change the strength of coupling with plasmons for all the folded modes. On the other hand, the coupling strength should decrease by increasing the reduced momentum *x_i_* of the mode since the plasmons are electronic collective excitations which are influenced not as much by the local structure and more likely take the zero reduced momentum. Based on these considerations, we suggest the following expression for the coupling strengths:

(4) $$\eta_{LO(TO),i}=\eta_{0}q_{LO(TO)}(1-\lambda_{LO(TO)}x_{i})$$

where the factor *η*_0_ represents the effect common to all modes, *q_LO_*_(*TO*)_ reflects the difference between the FLO and FTO modes, and *λ_LO_*_(*TO*)_ expresses the decreasing rate of the coupling strength by increasing the reduced momentum.

**Figure 5:**
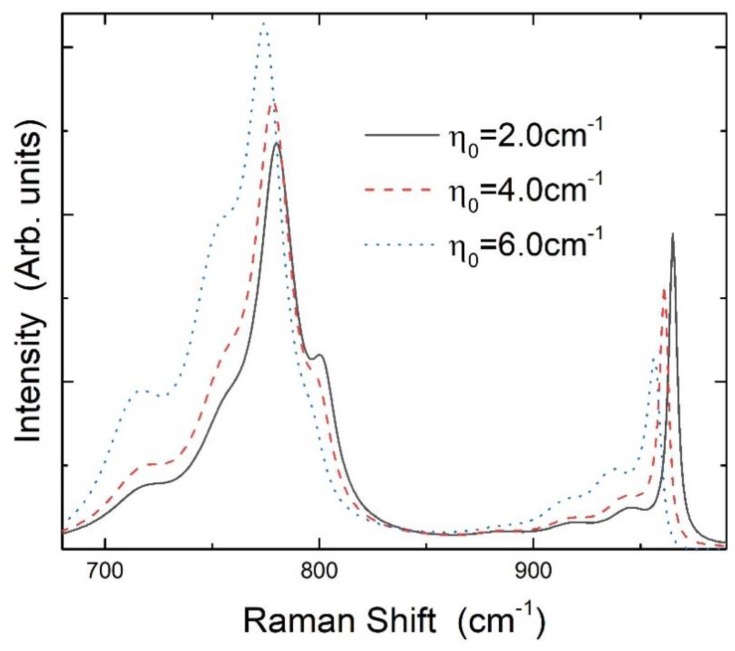
Calculated Raman spectra of the FTO and FLO modes for different values of common coupling strength *η*_0_. The parameters specifying the difference of the modes in coupling with the plasmons are as follows: $q_{LO}=4.5$, $q_{TO}=2.0$, $\lambda_{LO}=0.86$, and $\lambda_{TO}=0.31$. The damping rates for the FTO and FLO modes are: $\Gamma_{l,1}=5\;{\mathrm{cm}}^{-1}$, $\Gamma_{l,2}=25\;{\mathrm{cm}}^{-1}$, $\Gamma_{l,3}=30\;{\mathrm{cm}}^{-1}$, $\Gamma_{l,4}=35\;{\mathrm{cm}}^{-1}$, $\Gamma_{t,1}=15\;{\mathrm{cm}}^{-1}$, $\Gamma_{t,2}=20\;{\mathrm{cm}}^{-1}$, $\Gamma_{t,3}=33\;{\mathrm{cm}}^{-1}$, and $\Gamma_{t,4}=38\;{\mathrm{cm}}^{-1}$. For the plasmons, the frequency is $\omega_{e}=20\;{\mathrm{cm}}^{-1}$ and the damping rate is *γ* = 100 cm^−1^.

Using the above equation, one can solve the polarizations for given effective driving fields and calculate the Raman spectra (Supplementary Information). Figure 5 shows the results of the four FTO and four FLO modes for different values of *η*_0._ By increasing the film thickness or decreasing the MNC size to increase common coupling strength *η*_0_, downshifts occur from almost all the folded modes. The amounts of the four FTO modes are almost the same, but the degree of the downshifts of the FLO modes is evidently different due to the different coupling strengths with plasmons. We note that the experimentally measured line widths and relative heights of peaks of FTO and FLO modes can be fitted well by mainly adjusting the damping rates, $\Gamma_{t\left(l\right),i}$, which result from the de-coherence effects of the environment (all other hot excited phonons) on the corresponding modes and are not related to the downshifts of the modes by increasing the coupling with plasmons.

## 4. Materials and Methods

The 6H-SiC (1.25 μm in diameter, 99.8%) MNCs were purchased from Alfa Aesar and the 6H-SiC (400–600 nm in diameter, 99.9%) MNCs from Aladdin. In the typical synthesis, the Si substrate covered by 300 nm SiO_2_ was rinsed with water, acetone, and ethanol for 10 min each sequentially. After air drying, 0.05 g of the 6H-SiC powder were dissolved in 5 mL of CH_3_CH_2_OH and ultrasonically treated for 5 min. To prepare the SiC granular films with different thicknesses, different amounts of the solution were put on the substrate. The SiC films were formed in an oven at 60 °C for 10 min, followed by squeezing with a glass sheet to make them flat.

The 6H-SiC films were characterized by scanning electron microscopy (SEM, QUANTA 250FEG, Burlington, MA, USA), transmission electron microscopy (HR-TEM, JEOL-2100, Beijing, China) and XRD (Philips X’Per Pro X-ray diffractometer, Amsterdam, The Netherlands). The MNC size distribution was determined by software (Nano Measurer, Shanghai, China) based on the SEM and TEM images of the films. Good agreement was achieved between the SEM and TEM results. The film thickness was measured by optical surface profilometry (Zeta-20). UV-VIS-NIR absorption was conducted on the JH7600S spectrophotometer and Raman scattering was performed on a T64000 triple Raman system with a backscattering geometry equipped with a 514.5 nm argon laser as excitation source. The resolution of the spectrometer was 0.5 cm^−1^ and all the experiments were carried out at room temperature.

## 5. Conclusions

In summary, 6H-SiC granular films with different MNC sizes and film thicknesses show all the FTO and FLO phonon modes. The FTO and FLO modes show clear downshifts with increasing film thicknesses and decreasing MNC sizes. It is attributed to the change in the coupling between the folded mode and plasmons of the 6H-SiC MNCs. Our theoretical calculation indicates that when all the LO and TO modes are coupled with the plasmon mode, the Raman peaks indeed downshift. The coupling strengths of the FTO and FLO modes are different due to the different spatial structures of polarizations, but there are controllable parameters such as the MNC size and film thickness which can influence the common coupling strength of FTO and FLO modes.

## Figures and Tables

**Table 1:** Complete FTO and FLO mode frequencies in which the partial peak frequencies are derived by Lorenzian fitting (Figures S2–S7 in Supporting Information).

Mean Size	1.25 μm				0.55 μm	
Thickness (μm)	0.5	1.5	2.6	3.6	1.5	3.6
FTO(1)	765.0	760.0	756.0	750.0	757.5	747.5
FTO(2/3)	776.0	773.5	766.0	761.5	772.0	759.5
FTO(1/3)	786.5	782.0	776.5	772.0	780.0	770.0
FTO(0)	795.5	790.0	784.0	782.5	788.0	780.5
FLO(1)	881.5	882.0	883.5	885.5	880.0	881.0
FLO(2/3)	926.5	924.5	922.5	920.0	921.0	913.5
FLO(1/3)	944.5	941.5	938.5	935.0	939.0	930.0
FLO(0)	966.5	960.5	955.5	950.5	958.0	946.0

## Supplementary Materials

The following are available online at http://www.mdpi.com/1420-3049/23/9/2296/s1, Calculation of Raman spectrum, Surface modification, Figure S1: Raman spectra of 6H-SiC with/without ethanol treatment, Figure S2–S7: Raman spectra and Lorenzian fitted peaks.
