# Supplementary material for: Strong Coupling of Folded Phonons with Plasmons in 6H-SiC Micro/Nanocrystals
Source: Molecules. 2018 Sep 8;23(9):2296. doi: 10.3390/molecules23092296 (PMC6225379; doi:10.3390/molecules23092296)
Supplement: Supplementary file 1 [file molecules-23-02296-s001.pdf]

Supporting Information

Strong Coupling of Folded Phonons with Plasmons in 6H-SiC Micro/Nanocrystals

Yao Huang [1], Run Yang [1,*], Shijie Xiong [1], Jian Chen [2], and Xinglong Wu [1,*]

[1] National Laboratory of Solid State Microstructures and School of Physics, Nanjing University, Nanjing 210093, P. R. China; 550549045@qq.com; sjxiong@nju.edu.cn;

[2] National Laboratory of Solid State Microstructures and Research Institute of Superconductor Electronics, Nanjing University, Nanjing 210093, China; chenj63@nju.edu.cn

* Correspondence: 1225536206@qq.com (R.Y); hkxlwu@nju.edu.cn (X.L.W); Tel.: 0086-25-83686303

Calculation of Raman spectrum

The effective driving fields $D_{LO(TO),i}$ transfer power to the medium as follows:

$$P = 2\omega V \operatorname{Im}\left(\sum_i (\mu_{LO,i} D_{LO,i}^{*} P_{LO,i} + \mu_{TO,i} D_{TO,i}^{*} P_{TO,i}) + \mu_e D_e^{*} P_e \right),$$

where $\mu_{LO(TO),i}$ and μ_e are weights contributed from the i th FLO (FTO) mode and plasmons, respectively, and V is the involved medium volume. In Raman scattering, the power scattered into a mode of the Raman field at frequency ω_{out} is related to P by $P_{out} = \omega_{out} P / \omega$. Hence, the power scattered into a solid angle $d\Omega$ and frequency range $d\omega_{out}$ is $dP_{out} = \frac{V_{out} n_{out}^3 \omega_{out}^2 P_{out}}{(2\pi c)^3} d\omega_{out} d\Omega$, where c is the light velocity, and n_{out} and V_{out} are the refractive index and volume of outgoing medium, respectively. The differential Raman efficiency has the relationship of:

$$\frac{d^2 R}{d\omega d\Omega} \propto \operatorname{Im}\left(\sum_i (\mu_{LO,i} D_{LO,i}^{*} P_{LO,i} + \mu_{TO,i} D_{TO,i}^{*} P_{TO,i}) + \mu_e D_e^{*} P_e \right).$$

Accordingly, together with the solved polarizations from equations in the main text for the given effective driving fields, the relative Raman intensities can be calculated as a function of frequencies.

Surface modification

After ethanol treatment, it can be seen that the Raman spectrum after treatment has two new peaks, at 776 and 927.5 cm^{-1} respectively, and the Raman peaks at 882.5 cm^{-1} have been significantly improved compared to the untreated sample. The 776 cm^{-1} Raman peak should belong to the FTO (2/3) mode and the 927.5 cm^{-1} Raman peak should belong to the FLO (2/3) mode. It can be seen that by the surface modification of

6H-SiC, all the eight 6H-SiC FTO modes and FLO modes predicted by the theory appear, which may be associated with the introduction of ethanol to the hybridization of 6H-SiC electrons from SP3 orbits to SP2 orbitals, and a more active pi bond electrons will form plasmons under laser excitation. And at last leads to the plasmon phonon coupling, which enhanced the intensities of weak modes.

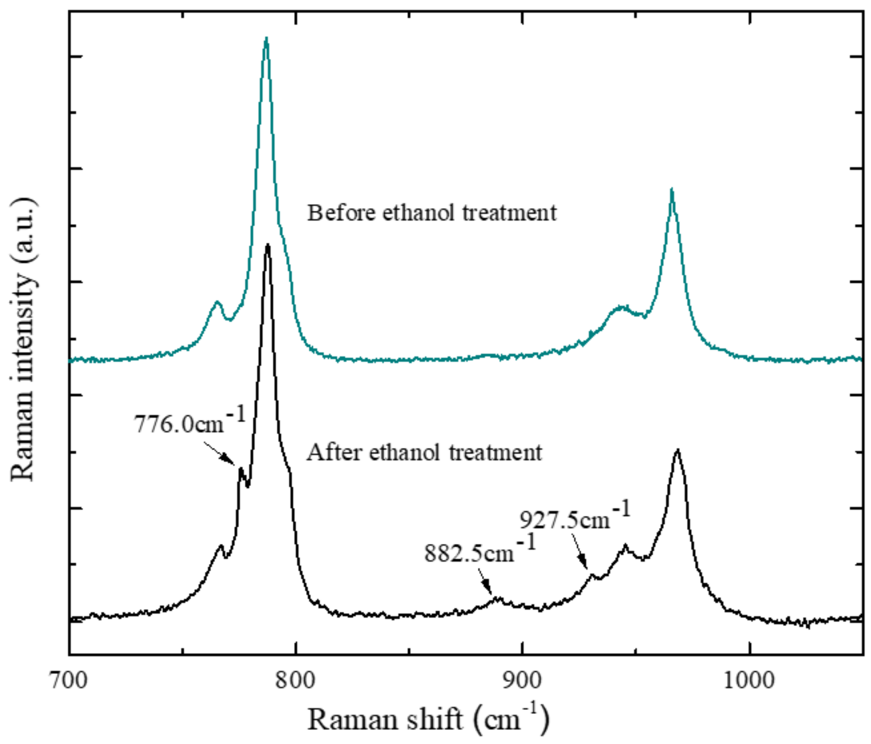

Figure S1. Comparison of Raman spectra of 6H-SiC with/without ethanol treatment.

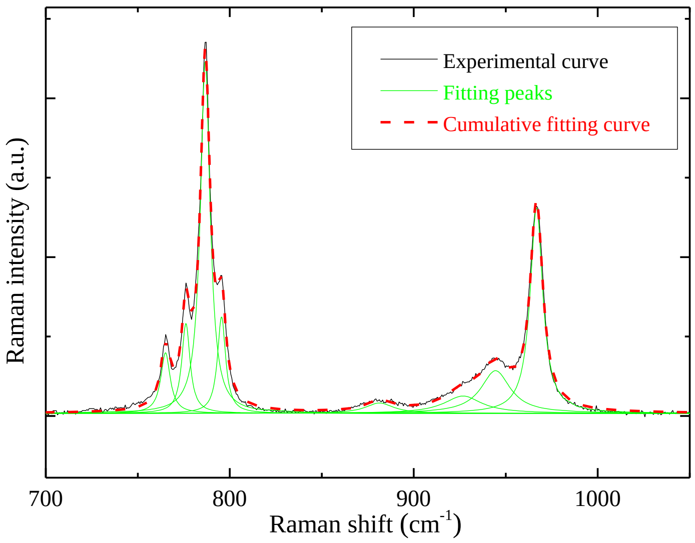

Figure S2. Raman spectra and Lorenzian fitted peaks of the 0.5 μm thick film with a mean size of 1.25 μm.

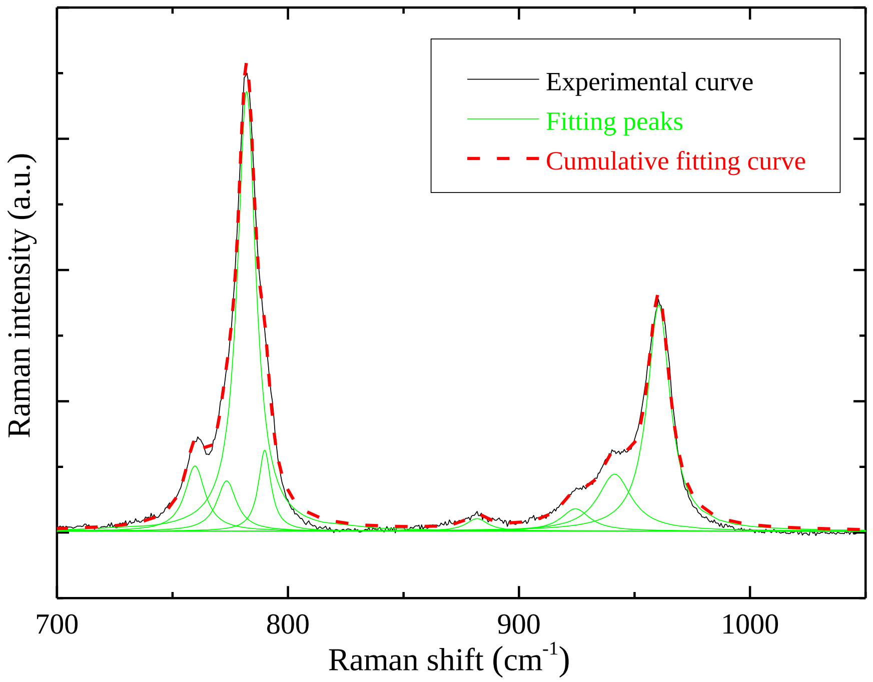

Figure S3. Raman spectra and Lorenzian fitted peaks of the 1.5 μm thick film with a mean size of 1.25 μm.

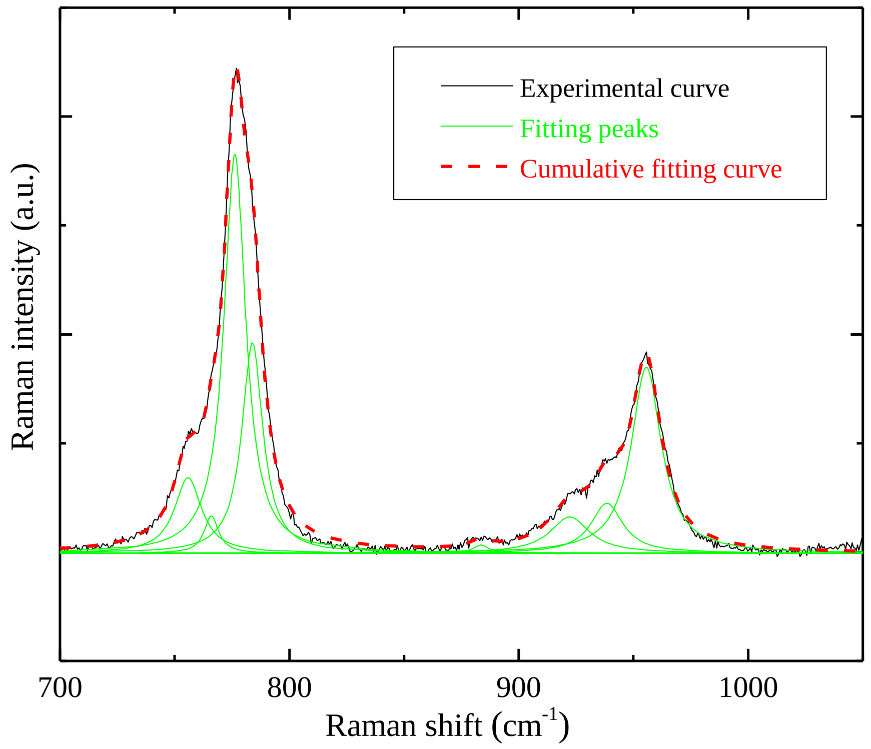

Figure S4. Raman spectra and Lorenzian fitted peaks of the 2.6 μm thick film with a mean size of 1.25 μm.

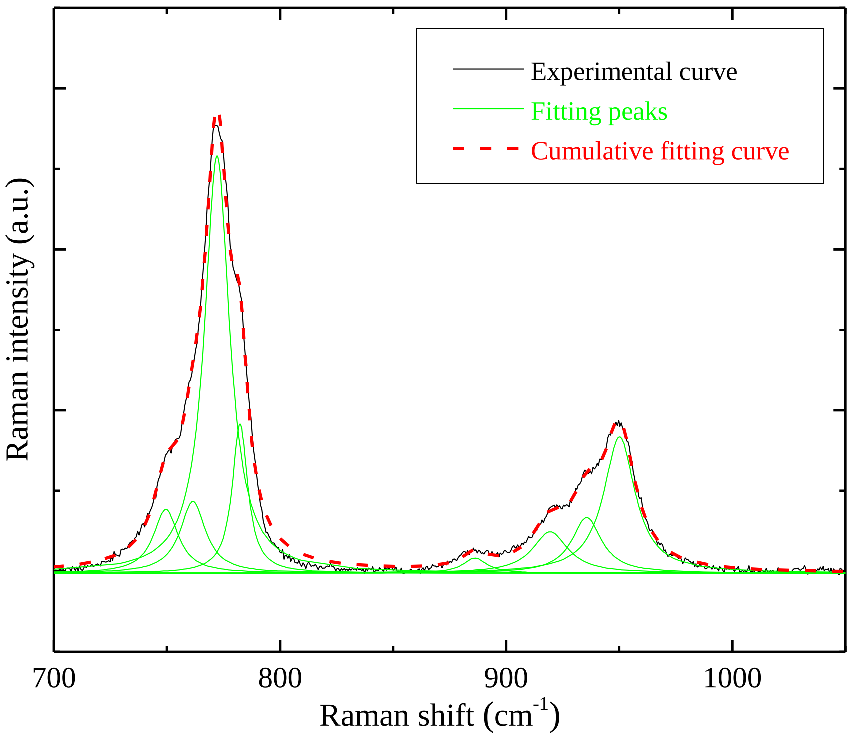

Figure S5. Raman spectra and Lorenzian fitted peaks of the 3.6 μm thick film with a mean size of 1.25 μm.

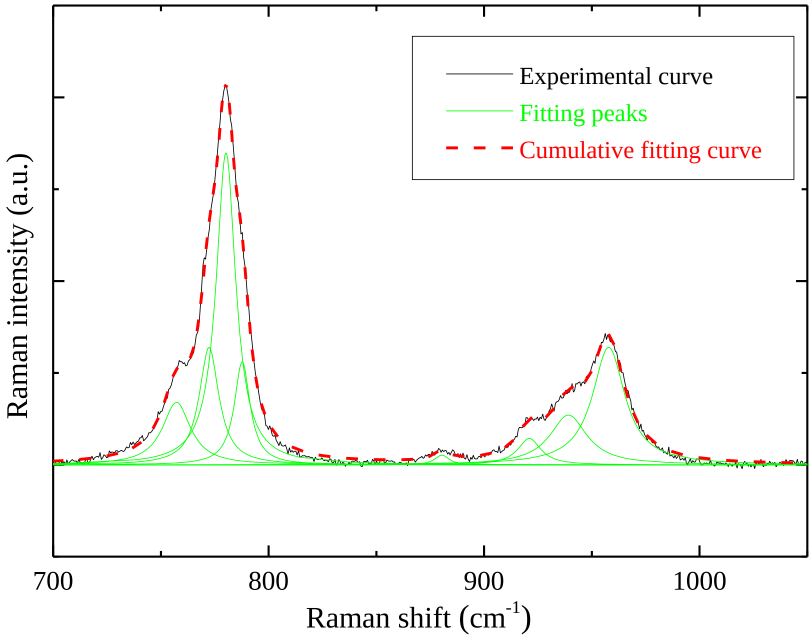

Figure S6. Raman spectra and Lorenzian fitted peaks of the 1.5 μm thick film with a mean size of 0.55 μm.

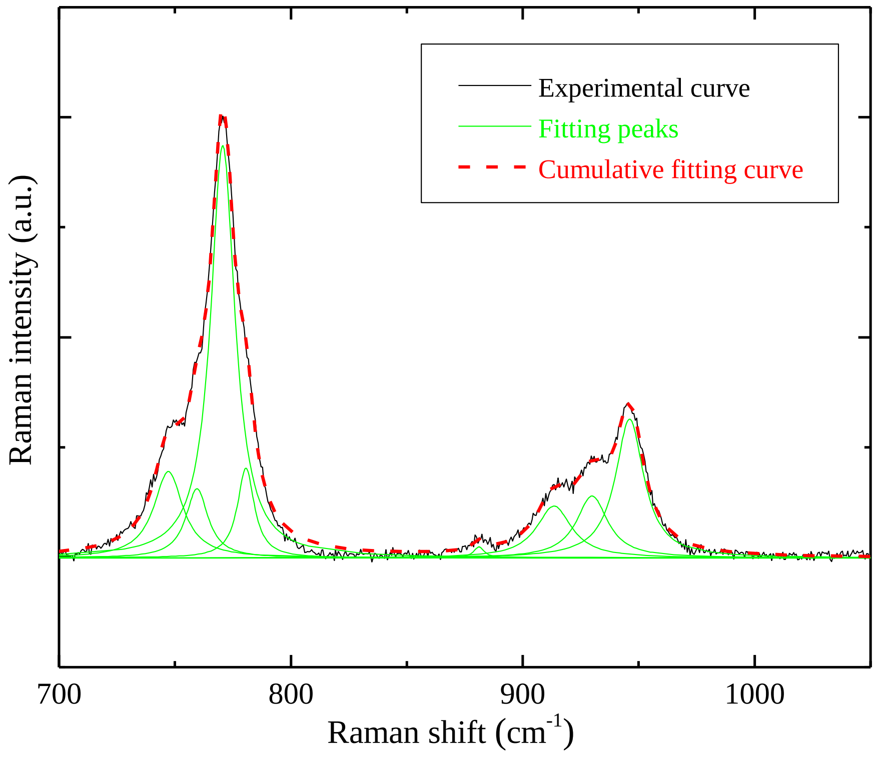

Figure S7. Raman spectra and Lorenzian fitted peaks of the 3.6 μm thick film with a mean size of 0.55 μm.
